# Redox Regulating Enzymes and Connected MicroRNA Regulators Have Prognostic Value in Classical Hodgkin Lymphomas

**DOI:** 10.1155/2017/2696071

**Published:** 2017-03-09

**Authors:** Peeter Karihtala, Katja Porvari, Ylermi Soini, Kirsi-Maria Haapasaari

**Affiliations:** ^1^Department of Oncology and Radiotherapy, Medical Research Center Oulu, Oulu University Hospital and University of Oulu, Oulu, Finland; ^2^Department of Pathology, Medical Research Center Oulu, Oulu University Hospital and University of Oulu, Oulu, Finland

## Abstract

There are no previous studies assessing the microRNAs that regulate antioxidant enzymes in Hodgkin lymphomas (HLs). We determined the mRNA levels of redox regulating enzymes peroxiredoxins (PRDXs) I–III, manganese superoxide dismutase (MnSOD), nuclear factor erythroid-derived 2-like 2 (Nrf2), and Kelch-like ECH-associated protein 1 (Keap1) from a carefully collected set of 41 classical HL patients before receiving any treatments. The levels of redoxmiRs, miRNAs known to regulate the above-mentioned enzymes, were also assessed, along with CD3, CD20, and CD30 protein expression. RNAs were isolated from freshly frozen lymph node samples and the expression levels were analyzed by qPCR. mir23b correlated inversely with CD3 and CD20 expressions (*p* = 0.00076; *r* = −0.523 and *p* = 0.0012; *r* = −0.507) and miR144 with CD3, CD20, and CD30 (*p* = 0.030; *r* = −0.352, *p* = 0.041; *r* = −0.333 and *p* = 0.0032; *r* = −0.47, resp.). High MnSOD mRNA levels associated with poor HL-specific outcome in the patients with advanced disease (*p* = 0.045) and high miR-122 levels associated with worse HL-specific survival in the whole patient population (*p* = 0.015). When standardized according to the CD30 expression, high miR212 and miR510 predicted worse relapse-free survival (*p* = 0.049 and *p* = 0.0058, resp.). In conclusion, several redoxmiRs and redox regulating enzyme mRNA levels associate with aggressive disease outcome and may also produce prognostic information in classical HL.

## 1. Introduction

Although modern polychemotherapy has significantly improved Hodgkin lymphoma (HL) survival rates, relapsed and refractory HL have still poor prognosis [1]. There are prognostic risk factor scores separately both in limited (IA–IIA) and in advanced (IIB–IV) stages of HL to guide the treatment selection. Nevertheless, prognosis varies considerably inside these classes and it would be of utmost importance to find novel prognostic and predictive biomarkers to help to select the patients at the highest risk for relapse.

We recently demonstrated a potential prognostic role of the protein expression of several cellular redox state regulating enzymes [2]. Since reactive oxygen species (ROS), especially superoxide anions, are produced primarily in mitochondria, mitochondrially located manganese superoxide dismutase (MnSOD or SOD2) is in the first-line defense by dismutating superoxide to the less harmful hydrogen peroxide (H_2_O_2_) and oxygen. H_2_O_2_ is further catalyzed to water and oxygen by peroxiredoxins (PRDXs), glutathione peroxidases, and catalase. PRDXs are a peroxidase family having multiple subcellular locations such as cytosol (PRDXs I, II, III, V, and VI), peroxisomes (PRDXs IV and V), lysosomes (PRDXs IV and VI), endoplasmic reticulum, extracellular space, Golgi apparatus (PRDX IV), and also mitochondria (PRDXs III) [3–6]. Their structure is conserved from bacteria to mammals and knockdown mice with PRDX I or II deficiency are susceptible to early aging and carcinogenesis [7–9]. PRDXs are involved in a variety of activities where H_2_O_2_ is generated, including cellular metabolism, growth, differentiation, inflammation, and proliferation [10].

Nuclear factor erythroid-derived 2-like 2 (Nrf2) is considered as the main cellular redox state regulator. It is a transcription factor able to regulate the expression of genes for antioxidant enzymes, metal-binding proteins, drug-metabolizing enzymes, drug transporters, and molecular chaperones. Under basal conditions, Nrf2 is bound to a cytoplasmic complex with its inhibitor Kelch-like ECH-associated protein 1 (Keap1). During oxidative stress Nrf2 is released and moves to the nucleus where it complexes with a small maf proteins and consequently upregulates the above-mentioned genes [11, 12].

MicroRNAs are small, single-stranded, noncoding RNAs that are essential posttranscriptional regulators of gene expression. The knowledge of miRNA in the regulation of redox state regulating enzymes (“redoxmiRs”) is rapidly increasing [13]. Although miRNAs are recognized as essential cellular homeostasis regulators, there is only limited amount of data on their role in the pathogenesis of HL with no existing literature on the impact of redoxmiRs in HL.

Cluster of differentiation proteins CD3, CD20, and CD30 are typically used as cell type markers of lymphocytes. Interestingly, they are membrane proteins with important physiological functions and likely to interact in oxidative stress also: CD3 is a coreceptor of T cell receptor, responsible for ligand-dependent initiation of intracellular signaling [14]. CD20 is a potential mediator of apoptosis through its interaction with membrane microdomains rich in src-family kinases [15], while CD30 is a member of tumor necrosis factor receptor superfamily [16].

Based on the results of our previous work assessing antioxidant protein expression in HL [2], we aimed here to clarify whether major redox regulator mRNA levels or miRNAs regulating them would associate with traditional prognostic factors of HL or chemoresistance or if they could serve as potential novel prognostic factors in classical HLs. We also compared their expression with CD30, a marker to detect Hodgkin's cells in classical Hodgkin's lymphoma, to CD20, a marker for B cell lineage, and CD3, a marker for T cell lineage.

## 2. Materials and Methods

### 2.1. Cases

The cohort consisted of 41 previously untreated classical Hodgkin lymphomas [17]. The samples were collected from the archives of Department of Pathology of Oulu University Hospital and they dated from the year 2000 until the year 2011. Freshly frozen lymph node samples were stored at −80°C. Clinical data of the patients were retrospectively collected from the patient records of Oulu University Hospital. Median follow-up of patients was 77.0 months and 6 patients had a relapse during the follow-up. Patients' characteristics are described in Table 1.

### 2.2. CD3, CD20, and CD30 Immunohistochemistry

Sections of 4 *µ*m thickness were cut from samples which had been routinely fixed in formalin and embedded in paraffin. The paraffin tissue sections were deparaffinized in xylene (5 min, 2 times) and rehydrated through graded ethanol. Antigen retrieval was performed by immersing sections in 10 mM sodium citrate buffer (pH 6.0) and boiling by microwave (95°C, 30 min). After boiling the sections were allowed to cool at room temperature and washed by PBS (5 min, 3 times). The sections were incubated in 3% hydrogen peroxidase for 10 min to inactivate endogenous peroxidases. After washing by PBS (5 min, 3 times), sections were incubated with the primary antibodies for 30 minutes at room temperature with a dilution of 1 : 300 for CD30 (code K5007, Dako, Dakopatts, Denmark), 1 : 1000 for CD20 (clone L26, Dako), and 1 : 50 for CD3 (clone PS1, Novocastra Biosystems Newcastle Ltd., Newcastle upon Tyne, UK). The following day, sections were washed repeatedly by PBS and incubated with Dako REALTM EnVision™/HRP, Rabbit/Mouse (Dako REAL EnVision Detection System: Dako, code K5007) for 1 h at room temperature. After washing repeatedly by PBS, the labelled secondary antibody was visualized by adding Dako REAL Substrate Buffer (Dako REAL EnVision Detection System: Dako, code K5007) containing Dako REAL DAB+ Chromogen (Dako REAL EnVision Detection System: Dako, code K5007). Sections were then counterstained with hematoxylin, dehydrated, and mounted. The data was evaluated by assessing the percentage of positive cells in the section for each antibody.

### 2.3. RNA Isolation and qPCR Analyses

Total RNA was extracted from lymph nodes using miRNeasy Mini Kit (Qiagen, Hilden, Germany). RNA quality was checked by electrophoresis. cDNA synthesis was done with miScript II Reverse Transcription Kit (Qiagen). miScript SYBR Green PCR Kit (Qiagen) was used for cDNA amplification by Rotor-Gene Q real-time quantitative PCR equipment (Qiagen). The following gene-specific primers were used for amplification; Keap1 (NM_203500.1): F (forward primer): 5′-GGTCCCCTACAGCCAAGGT-3′ and R: (reverse primer) 5′-CTGCATGGGGTTCCAGAAGAT-3′; Nrf2 (S74017.1): F: 5′-GCAGGACATGGATTTGATTGACA-3′ and R: 5′-TCATACTCTTTCCGTCGCTGA-3′; PRDX1 (NM_002574.3): F: 5′-CACTGACAAACATGGGGAAGT-3′ and R: 5′-TGCTCTTTTGGACATCAGGCT-3′; PRDX2 (NM_005809.5): F: 5′-GTCCAGGCCTTCCAGTACAC-3′ and R: 5′-TGTCATCCACGTTGGGCTTA-3′; PRDX3 (NM_006793.3): F: 5′-TTAAACATGGTTAGTTGCTAGTACAAGGA-3′ and R: 5′-TTGAGACATGATCTAAGAATAGCCTTCTA-3′; MnSOD (NM_000636.2): F: 5′-GGACACTTACAAATTGCTGCTT-3′ and R: 5′-CCACACATCAATCCCCAGCA-3′. GAPD (BC029618; F: 5-TGGAAGGACTCATGACCACA-3′ and R: 5-CCATCACGCCACAGTTT-3′) was used as reference gene for normalization of qPCR results in mRNA studies. For miRNA quantification, the following commercial miScript Primer Assays (Qiagen) were used: MIR23B, MIR28, MIR93, MIR122, MIR144, MIR200A, MIR212, MIR383, and MIR510. miScript Primer Assay for RNU6B was used for normalization of qPCR results in miRNA studies. Cycling was carried out as recommended in the PCR Kit with annealing temperatures of 60–68 or 55°C for mRNA and miRNA, respectively. Fluorescence signals were measured continuously during repetitive cycles in order to detect Ct values for target RNA and reference (GAPD or RNU6B) in the samples. Relative expression levels of mRNA or miRNA targets were calculated using 2^−ΔΔCt^ method [18], where ΔΔCt = (Ct_target  RNA_ − Ct_GAPD  or  RNU6B_)_sample_ − (Ct_target  RNA_ − Ct_GAPD  or  RNU6B_)_reference  sample_.

### 2.4. Statistical Analyses

Statistical analyses were performed by using IBM SPSS Statistics software, v. 22.0.0.0 (IBM Corporation, Armonk, NY, USA). The significance of associations was defined by using Mann–Whitney *U* test and Spearman's rho test with correlation coefficient. Kaplan–Meier curves with the log-rank test were applied in survival analysis. In survival analyses, median mRNA and miRNA levels were used as a cut-off for two-classed variable. Disease-specific survival (DSS) was calculated from the time of diagnosis to the time of confirmed Hodgkin lymphoma-related death and relapse-free survival (RFS) from the time of diagnosis to the confirmed relapse. Values of *p* of less than 0.05 were considered significant.

The ethical committee of the Northern Ostrobothnia Hospital District approved the study design. During the data collection and management, the principles of the Declaration of Helsinki were followed.

## 3. Results

CD3, CD20, and CD30 immunohistochemistry were applied to 37 (90.2%) samples. The median expressions of CD3, CD20, and CD30 were 65%, 35%, and 7%, and the ranges of expression were 30–95%, 5–80%, and 1–55%, respectively.

CD3 expression correlated with CD20 expression highly significantly (*p* = 0.000040; *r* = 0.615). mir23b correlated inversely with CD3 and CD20 expressions (*p* = 0.00076; *r* = −0.523 and *p* = 0.0012; *r* = −0.507) and miR144 with CD3, CD20, and CD30 (*p* = 0.030; *r* = −0.352, *p* = 0.041; *r* = −0.333 and *p* = 0.0032; *r* = −0.47, resp.). These and other correlations between CD3, CD20, and CD30 and redoxmiRs and mRNAs are presented in Table 2.

Higher redox regulator mRNA levels associated consistently with the traditional factors of poor prognosis (Table 3). PRDX I mRNA expression associated with the presence of limited stage risk factors (*p* = 0.027) and was the most highly expressed in nodular sclerosis subtype, following mixed cell histology, and the least expressed in the tumors with classical lymphocyte-rich subtype (*p* = 0.008). MnSOD mRNA expression associated with the presence of B-symptoms (*p* = 0.006) and higher Nrf2 mRNA levels with the presence of risk factors of limited disease (*p* = 0.027). Nrf2 and Keap1 mRNA expressions correlated strongly with each other (*p* = 1.09 × 10^−9^, *r* = 0.787).

miR-383 (*p* = 0.019) and miR-200a (*p* = 0.044) were the most frequently expressed in the tumors with nodular sclerosis histology, following mixed cell histology. It was least expressed in the tumors with classical lymphocyte-rich subtype. miR-383 also associated with the presence of advanced disease (*p* = 0.014) and B-symptoms at the time of diagnosis (*p* = 0.013). Likewise, miR-23b associated with advanced stage (*p* = 0.021) and B-symptoms (*p* = 0.025). miR-212 and miR-28 levels were elevated in advanced stage tumors (*p* values 0.047 and 0.036, resp.). High miR-122 expression associated with the presence of B-symptoms.

Higher MnSOD mRNA levels associated with shorter disease-specific survival (DSS), but only in the patients with advanced disease (*p* = 0.045). miR-122 levels over median were also in association with poor DSS (*p* = 0.015) in the whole patient population.

**Figure 1:**
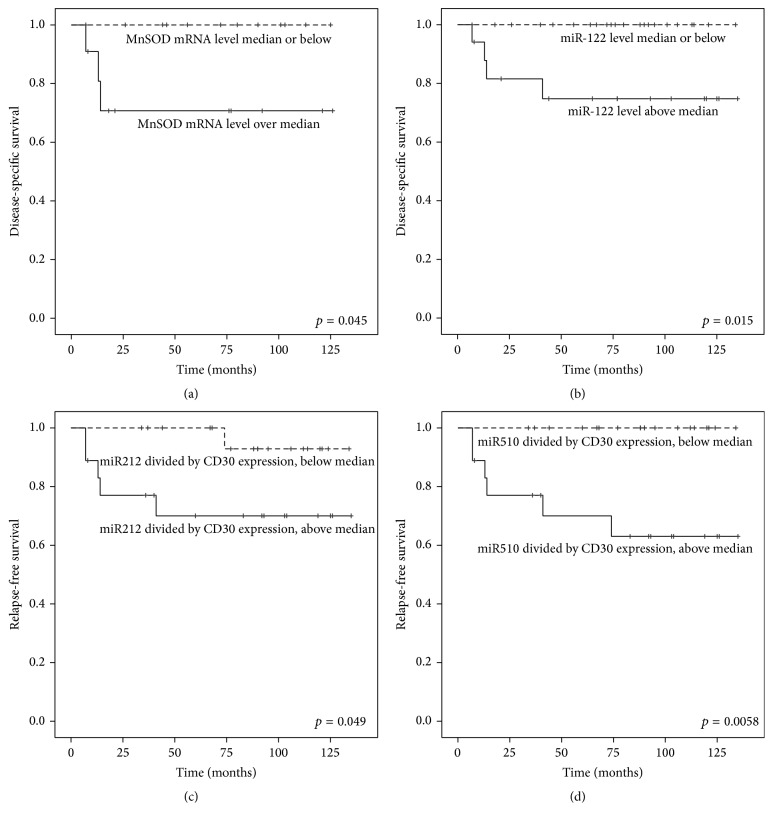
Kaplan–Meier curve of disease-specific survival according to MnSOD mRNA level only in patients with advanced stage (a) and miR-122 level in the whole patient population (b). Relapse-free survival is demonstrated when miR212 (c) and miR510 (d) are standardized according to the CD30 expression.

When the results were standardized according to the CD30 expression (miRNA or mRNA variable divided by CD30 expression), there were no significant associations with stage, histology, chemotherapy response, or risk factors. Instead, higher miR212 and miR510 levels predicted worse RFS (*p* = 0.049 and *p* = 0.0058, resp.) (Figure 1).

## 4. Discussion

We report here a potential role of high mitochondrial antioxidant enzyme MnSOD mRNA levels in predicting worse prognosis of patients with untreated classical HL. This is in line with our previous study assessing MnSOD protein expression in HL [2] and may be related to the role of MnSOD in chemoresistance. In addition, miR510, PRDX I suppressing redoxmiR, was a highly significant predictor of dismal RFS when miR510 level was related to CD30 protein expression.

The most common first-line treatment of classical HL is ABVD, consisting of intensive dosing of doxorubicin, bleomycin, vinblastine, and dacarbazine. The mechanism of action of these drugs is closely linked to ROS generation, especially in the case of bleomycin. Although doxorubicin and vinblastine have other mechanisms of action, they also induce ROS generation leading to enhanced oxidative stress and eventually apoptosis [19, 20]. Dacarbazine produces also toxic amounts of ROS contributing to cell lysis [21]. Increasing evidence suggests that antioxidant enzyme levels may confer to chemoresistance in solid tumors, although evidence is still scarce in lymphomas [22, 23] and almost absent in HLs. We previously reported that stronger immunohistochemical MnSOD expression is a predictor of worse RFS in classical HLs. Consistently, in the current material MnSOD mRNA levels associated with poor DSS rates. This was observed only with the patients with advanced disease, among the patient group most eagerly requiring novel prognostic and predictive factors to guide treatment intensification. miR-212 has been identified to interact with MnSOD mRNA and miR-212 downregulation is able to promote colorectal cancer metastasis growth in animal models [24]. This effect seems to be cancer-specific since in the current material increased miR-212 level was associated with advanced rather than limited stage. High miR212 levels also predicted worse RFS, when the expression was related to CD30 expression.

Both plasma miRNA levels and specific miRNA signatures have been demonstrated to have prognostic significance in classical HL [25, 26]. Although there are no previous publications on the miRNA regulation of redox status in HL, there is a plenty of data on the impact of miRNAs to redox regulators in other cancers. miR-28, miR-144, and miR-93 have been shown to directly repress the major antioxidant response inducer Nrf2 mRNA [27]. On the other hand, there is emerging evidence that Nrf2 can regulate the expression of various miRNAs [28]. Nrf2 has emerged as a prognostic factor in multiple solid carcinomas [29–31] and the blockage of Nrf2 by siRNA leads to the inhibition of tumor growth and increased chemosensitivity [32]. We found that Nrf2 mRNA levels associated with the presence of traditional risk factors of limited stage HL and miR-28 was clearly overexpressed in the advanced disease. Although the results with miR-28 are more difficult to explain with the current knowledge of its function, our data suggests that Nrf2 may accelerate HL lymphomagenesis. This would be in line with multiple solid carcinomas, but, to confirm this hypothesis, more mechanistic approach should be adopted in further studies. In addition, we noted that Keap1 and Nrf2 mRNA levels had extremely significant correlation. Although this seems logical based on the inhibitory function of Keap1 against Nrf2 activation, to the best of our knowledge this correlation has been previously reported only at protein level, but not at mRNA level [33].

In solid tumors PRDX overexpression has been connected to chemoresistance and radiotherapy resistance, the best data being available from PRDX II [34, 35]. There is increasing evidence from in vitro studies that PRDX II may block especially doxorubicin-mediated cell death [36, 37]. PRDX I and II overexpression have also been connected to poorer prognostic factors in several carcinomas [36, 38–40]. In the present study PRDX I associated with more aggressive histological subtypes of classical HL and the presence of risk factors in limited stage patients. Although these are surrogates to aggressive disease course in general, no link between PRDX I mRNA expression and survival or chemoresistance was observed. miR510 has been proposed as PRDX I 3′UTR binding, tumor growth promoting oncomiR in breast cancer [41, 42], while in ovarian cancer high miR510 expression levels associate with better outcome [43]. When we standardized miR510 expression with CD30 expression, none of the patients with low mir510 expression had relapse during the follow-up, compared to the dismal RFS of 63% at 6 years in those with high mir510 levels. Furthermore, high levels of PRDX II mRNA targeting miRNA, miR-122 [44], associated with the dismal outcome in the current material. The prognostic role of miR-122 has been previously quite extensively studied especially in hepatocellular carcinoma with both worse and better survival being reported [45–47].

miR-23b and miR-383 have been recognized to downregulate the mitochondrial isoform of PRDX family, PRDX III [48, 49]. miR-23b and miR-383 were linked to advanced stage and B-symptoms and miR-383 associated also with poor risk factor profile of limited disease and to more aggressive histological subtype. miR-23b is a well characterized miRNA of aggressive features of several solid carcinomas (“oncomiR”) and may induce tumor survival, accelerate glioma invasion, and promote prostate cancer cell proliferation by regulating PTEN and its downstream signaling [50, 51]. In gastric cancer miR-23b associated with unfavorable survival [52]. The existing evidence from miR-383 suggests its tumor-suppressing role in vitro, for example, hepatocellular, pancreatic, and esophageal carcinomas [53–55]. This is in contradiction with our results, which suggested both miR-23b and miR-383 act clearly oncogenic in Hodgkin lymphomas.

CD30 is a well-known marker for Hodgkin's cells in classical Hodgkin's lymphoma. Hodgkin's lymphoma is a type of neoplastic disease where the tissue contains abundant reactive elements. Of lymphocytes T cells are overwhelmingly present but B cells and macrophages constitute also a significant part of the reactive elements in tumor tissue. No reports have been published on CD30 and miRNAs studied here. In ALK negative large anaplastic lymphomas there was a high miR-155 expression [56]. Interestingly, miR-155 is also highly expressed in Hodgkin lymphoma cell lines, but CD30 is not a target for this miRNA even though anaplastic large cell lymphoma and Hodgkin lymphoma both share its expression [57]. Additionally, miR-155 is elevated in all B cell lymphomas except Burkitt's lymphoma [58].

CD3 complex mediates activation of the T cell receptor across the plasma membrane [59]. Since T cells are most abundantly expressed in Hodgkin lymphoma low miR-23b or miR-144 expression would be expected to be due to low miRNA synthesis in these cells. In scanning miRNA levels from blood cells these miRNAs were not, however, found to be present among the most over- or underexpressed miRNAs in CD3 or CD19 positive cells [60]. miR-23b or miR-144 are not either targets against CD3 gene complex [61]. The miRNAs studied here, however, are regulating the Nrf2/Keap1 axis in these cells, and in activated T or B cells their expression may change. The inverse association of CD3 as well as CD20 with miR-23b or miR-144 thus suggests a promotion of Nrf2 related antioxidative response in the lymphatic elements. In line with this, high miR-144 levels have been shown to reduce Nrf2 levels [62]. miR23, on the other hand, has an impact on Keap1 synthesis [63]. All in all, the associations between CD30, CD3, CD20, and the miRNAs shown in this study most likely reflects the activation of the oxidative defense system in stromal lymphocytes and Hodgkin cells.

## 5. Conclusions

This study gives support to the previous evidence that MnSOD expression could have prognostic impact in untreated advanced HL. It is possible that MnSOD mRNA levels could also be used as predictive factor, but this has to be assessed in future studies. Several redoxmiRs, especially miR510, also appear as potentially interesting indicators of aggressive disease course and should be validated in larger cohort of HLs, the most optimally with microdissection technique enabling assessing different tumor compartments separately.

## Figures and Tables

**Table 1:** Patient characteristics.

Male	20 (48.8%)
Limited stage	17 (41.5%)
Limited stage risk factors	8 (47.1%)^1^
International Prognostic Score > 2	9 (37.5%)^1^
B-symptoms	18 (43.9%)
WHO performance status ≥ 1	31.3%
Radiotherapy	21 (51.2%)
Relapsed	6 (14.6%)
CR with 1st-line ABVD	34 (82.9%)

^1^Percentage from the patients with limited stage and advanced stage, respectively.

**Table 2:** Associations between CD3, CD20, and CD30 protein levels and redoxmRNAs and miRNAs regulating them.

	CD3	CD20	CD30
CD3			
CD20	*p* = 0.000040; *r* = 0.615		
CD30	NS	NS	NS
PRDX I mRNA	*p* = 0.037; *r* = −0.339	NS	NS
PRDX II mRNA			
PRDX III mRNA		*p* = 0.046; *r* = −0.326	
Nrf2 mRNA			
Keap1 mRNA		*p* = 0.050; *r* = −0.320	
SOD2 mRNA			
miR23b	*p* = 0.00076; *r* = −0.523	*p* = 0.0012; *r* = −0.507	
miR28	*p* = 0.022; *r* = −0.370	*p* = 0.011; *r* = −0.409	
miR93		*p* = 0.031; *r* = −0.351	
miR122			
miR144	*p* = 0.030; *r* = −0.352	*p* = 0.041; *r* = −0.333	*p* = 0.0032; *r* = −0.471
miR200a			
miR212			
miR383			
miR510			

**Table 3:** Two-sided *p* values < 0.05 when the clinicopathological parameters are set against mRNA and miRNA expression. ↑ and ↓ indicate the direction of the correlation.

Expression of mRNA or miRNA	Histology	Stage		B-symptoms	Risk factors		Disease-specific survival	
		Limited	Advanced		Limited disease	Advanced disease	Limited disease	Advanced disease
PRDX I	0.008^1^				0.027			
miR-510								
PRDX II								
miR-122↑				0.037			0.015	
PRDX III								
miR-23b↑			0.021	0.025				
miR-383↑	0.019^1^		0.014	0.013	0.015			
MnSOD↑				0.006				0.045
miR-212↑			0.047					
Nrf2↑					0.027			
miR-28↑			0.036					
miR-93								
miR-144								
Keap1								
miR-200a↑	0.044^1^							

^1^Most frequently expressed in nodular sclerosis subtype followed by mixed cell histology and least expressed in lymphocyte-rich subtype.

## References

[1] Kuruvilla J., Keating A., Crump M. (2011). How I treat relapsed and refractory Hodgkin lymphoma. *Blood*.

[2] Bur H., Haapasaari K.-M., Turpeenniemi-Hujanen T. (2014). Oxidative stress markers and mitochondrial antioxidant enzyme expression are increased in aggressive Hodgkin lymphomas. *Histopathology*.

[3] Okado-Matsumoto A., Matsumoto A., Fujii J., Taniguchi N. (2000). Peroxiredoxin IV is a secretable protein with heparin-binding properties under reduced conditions. *The Journal of Biochemistry*.

[4] Declercq J.-P., Evrard C., Clippe A., Stricht D. V., Bernard A., Knoops B. (2001). Crystal structure of human peroxiredoxin 5, a novel type of mammalian peroxiredoxin at 1.5 Å resolution. *Journal of Molecular Biology*.

[5] Kinnula V. L., Lehtonen S., Sormunen R. (2002). Overexpression of peroxiredoxins I, II, III, V, and VI in malignant mesothelioma. *Journal of Pathology*.

[6] Kang S. W., Baines I. C., Rhee S. G. (1998). Characterization of a mammalian peroxiredoxin that contains one conserved cysteine. *Journal of Biological Chemistry*.

[7] Rani V., Neumann C. A., Shao C., Tischfield J. A. (2012). Prdx1 deficiency in mice promotes tissue specific loss of heterozygosity mediated by deficiency in DNA repair and increased oxidative stress. *Mutation Research—Fundamental and Molecular Mechanisms of Mutagenesis*.

[8] Cao J., Schulte J., Knight A. (2009). Prdx1 inhibits tumorigenesis via regulating PTEN/AKT activity. *EMBO Journal*.

[9] Han Y.-H., Kim H.-S., Kim J.-M., Kim S.-K., Yu D.-Y., Moon E.-Y. (2005). Inhibitory role of peroxiredoxin II (Prx II) on cellular senescence. *FEBS Letters*.

[10] Karihtala P., Soini Y. (2007). Reactive oxygen species and antioxidant mechanisms in human tissues and their relation to malignancies. *APMIS*.

[11] Itoh K., Mimura J., Yamamoto M. (2010). Discovery of the negative regulator of Nrf2, keap1: a historical overview. *Antioxidants and Redox Signaling*.

[12] Hu R., Saw C. L.-L., Yu R., Kong A.-N. T. (2010). Regulation of NF-E2-related factor 2 signaling for cancer chemoprevention: antioxidant coupled with antiinflammatory. *Antioxidants and Redox Signaling*.

[13] Cheng X., Ku C.-H., Siow R. C. M. (2013). Regulation of the Nrf2 antioxidant pathway by microRNAs: new players in micromanaging redox homeostasis. *Free Radical Biology and Medicine*.

[14] Pitcher L. A., Van Oers N. S. C. (2003). T-cell receptor signal transmission: who gives an ITAM?. *Trends in Immunology*.

[15] Deans J. P., Li H., Polyak M. J. (2002). CD20-mediated apoptosis: signalling through lipid rafts. *Immunology*.

[16] Schneider C., Hübinger G. (2002). Pleiotropic signal transduction mediated by human CD30: a member of the tumor necrosis factor receptor (TNFR) family. *Leukemia and Lymphoma*.

[17] Swerdlow S. H., Campo E., Harris N. L. (2008). Mature B-cell neoplasms. *WHO Classification of Tumours of Hematopoietic and Lymphoid Tissues*.

[18] Livak K. J., Schmittgen T. D. (2001). Analysis of relative gene expression data using real-time quantitative PCR and the 2(-Delta Delta C(T)) Method. *Methods*.

[19] Dorr R. T. (1992). Bleomycin pharmacology: mechanism of action and resistance, and clinical pharmacokinetics. *Seminars in Oncology*.

[20] Fang J., Nakamura H., Iyer A. K. (2007). Tumor-targeted induction of oxystress for cancer therapy. *Journal of Drug Targeting*.

[21] Pourahmad J., Amirmostofian M., Kobarfard F., Shahraki J. (2009). Biological reactive intermediates that mediate dacarbazine cytotoxicity. *Cancer Chemotherapy and Pharmacology*.

[22] Tome M. E., Frye J. B., Coyle D. L. (2012). Lymphoma cells with increased anti-oxidant defenses acquire chemoresistance. *Experimental and Therapeutic Medicine*.

[23] Kuusisto M. E. L., Haapasaari K.-M., Turpeenniemi-Hujanen T. (2015). High intensity of cytoplasmic peroxiredoxin VI expression is associated with adverse outcome in diffuse large B-cell lymphoma independently of International Prognostic Index. *Journal of Clinical Pathology*.

[24] Meng X., Wu J., Pan C. (2013). Genetic and epigenetic down-regulation of MicroRNA-212 promotes colorectal tumor metastasis via dysregulation of MnSOD. *Gastroenterology*.

[25] Sánchez-Espiridión B., Martín-Moreno A. M., Montalbán C. (2013). MicroRNA signatures and treatment response in patients with advanced classical Hodgkin lymphoma. *British Journal of Haematology*.

[26] Jones K., Nourse J. P., Keane C., Bhatnagar A., Gandhi M. K. (2014). Plasma microRNA are disease response biomarkers in classical hodgkin lymphoma. *Clinical Cancer Research*.

[27] Yang M., Yao Y., Eades G., Zhang Y., Zhou Q. (2011). MiR-28 regulates Nrf2 expression through a Keap1-independent mechanism. *Breast Cancer Research and Treatment*.

[28] Shah N. M., Rushworth S. A., Murray M. Y., Bowles K. M., MacEwan D. J. (2013). Understanding the role of NRF2-regulated miRNAs in human malignancies. *Oncotarget*.

[29] Solis L. M., Behrens C., Dong W. (2010). Nrf2 and Keap1 abnormalities in non-small cell lung carcinoma and association with clinicopathologic features. *Clinical Cancer Research*.

[30] Soini Y., Eskelinen M., Juvonen P. (2014). Nuclear Nrf2 expression is related to a poor survival in pancreatic adenocarcinoma. *Pathology Research and Practice*.

[31] Hartikainen J. M., Tengström M., Kosma V.-M., Kinnula V. L., Mannermaa A., Soini Y. (2012). Genetic polymorphisms and protein expression of NRF2 and sulfiredoxin predict survival outcomes in breast cancer. *Cancer Research*.

[32] Singh A., Boldin-Adamsky S., Thimmulappa R. K. (2008). RNAi-mediated silencing of nuclear factor erythroid-2-related factor 2 gene expression in non-small cell lung cancer inhibits tumor growth and increases efficacy of chemotherapy. *Cancer Research*.

[33] Huang C.-F., Zhang L., Ma S.-R. (2013). Clinical significance of keap1 and Nrf2 in oral squamous cell carcinoma. *PLOS ONE*.

[34] Wang T., Diaz A. J. G., Yen Y. (2014). The role of peroxiredoxin II in chemoresistance of breast cancer cells. *Breast Cancer: Targets and Therapy*.

[35] Smith-Pearson P. S., Kooshki M., Spitz D. R., Poole L. B., Zhao W., Robbins M. E. (2008). Decreasing peroxiredoxin II expression decreases glutathione, alters cell cycle distribution, and sensitizes glioma cells to ionizing radiation and H_2_O_2_. *Free Radical Biology and Medicine*.

[36] Kubota D., Mukaihara K., Yoshida A., Tsuda H., Kawai A., Kondo T. (2013). Proteomics study of open biopsy samples identifies peroxiredoxin 2 as a predictive biomarker of response to induction chemotherapy in osteosarcoma. *Journal of Proteomics*.

[37] McDonald C., Muhlbauer J., Perlmutter G., Taparra K., Phelan S. A. (2014). Peroxiredoxin proteins protect MCF-7 breast cancer cells from doxorubicin-induced toxicity. *International Journal of Oncology*.

[38] Suenaga S., Kuramitsu Y., Wang Y. (2013). Human pancreatic cancer cells with acquired gemcitabine resistance exhibit significant up-regulation of peroxiredoxin-2 compared to sensitive parental cells. *Anticancer Research*.

[39] Sun Q.-K., Zhu J.-Y., Wang W. (2014). Diagnostic and prognostic significance of peroxiredoxin 1 expression in human hepatocellular carcinoma. *Medical Oncology*.

[40] Kalinina E. V., Berezov T. T., Shtil' A. A. (2012). Expression of peroxiredoxin 1, 2, 3, and 6 genes in cancer cells during drug resistance formation. *Bulletin of Experimental Biology and Medicine*.

[41] Guo Q. J., Mills J. N., Bandurraga S. G. (2013). MicroRNA-510 promotes cell and tumor growth by targeting peroxiredoxin1 in breast cancer. *Breast Cancer Research*.

[42] Gaj P., Zagozdzon R. (2014). In silico analysis of microRNA-510 as a potential oncomir in human breast cancer. *Breast Cancer Research*.

[43] Zhang X., Guo G., Wang G. (2015). Profile of differentially expressed miRNAs in high-grade serous carcinoma and clear cell ovarian carcinoma, and the expression of miR-510 in ovarian carcinoma. *Molecular Medicine Reports*.

[44] Diao S., Zhang J.-F., Wang H. (2010). Proteomic identification of microRNA-122a target proteins in hepatocellular carcinoma. *Proteomics*.

[45] Cho H. J., Kim J. K., Nam J. S. (2015). High circulating microRNA-122 expression is a poor prognostic marker in patients with hepatitis B virus-related hepatocellular carcinoma who undergo radiofrequency ablation. *Clinical Biochemistry*.

[46] Xu Y., Bu X., Dai C., Shang C. (2015). High serum microRNA-122 level is independently associated with higher overall survival rate in hepatocellular carcinoma patients. *Tumor Biology*.

[47] Köberle V., Kronenberger B., Pleli T. (2013). Serum microRNA-1 and microRNA-122 are prognostic markers in patients with hepatocellular carcinoma. *European Journal of Cancer*.

[48] Li K. K., Pang J. C., Lau K. M. (2013). MiR-383 is downregulated in medulloblastoma and targets peroxiredoxin 3 (PRDX3). *Brain Pathology*.

[49] He H.-C., Zhu J.-G., Chen X.-B. (2012). MicroRNA-23b downregulates peroxiredoxin III in human prostate cancer. *FEBS Letters*.

[50] Tian L., Fang Y.-X., Xue J.-L., Chen J.-Z. (2013). Four microRNAs promote prostate cell proliferation with regulation of PTEN and its downstream signals in vitro. *PlOS one*.

[51] Chen L., Han L., Zhang K. (2012). VHL regulates the effects of miR-23b on glioma survival and invasion via suppression of HIF-1*α*/VEGF and *β*-catenin/Tcf-4 signaling. *Neuro-Oncology*.

[52] Ma G., Dai W., Sang A., Yang X., Gao C. (2014). Upregulation of microRNA-23a/b promotes tumor progression and confers poor prognosis in patients with gastric cancer. *International Journal of Clinical and Experimental Pathology*.

[53] Han S., Cao C., Tang T. (2015). ROBO3 promotes growth and metastasis of pancreatic carcinoma. *Cancer Letters*.

[54] Wang X., Ren Y., Wang Z. (2015). Down-regulation of 5S rRNA by miR-150 and miR-383 enhances c-Myc-rpL11 interaction and inhibits proliferation of esophageal squamous carcinoma cells. *FEBS Letters*.

[55] Chen L., Guan H., Gu C., Cao Y., Shao J., Wang F. (2016). miR-383 inhibits hepatocellular carcinoma cell proliferation via targeting APRIL. *Tumor Biology*.

[56] Merkel O., Hamacher F., Griessl R. (2015). Oncogenic role of MIR-155 in anaplastic large cell lymphoma lacking the t(2;5) translocation. *The Journal of Pathology*.

[57] Gibcus J. H., Tan L. P., Harms G. (2009). Hodgkin lymphoma cell lines are characterized by a specific miRNA expression profile. *Neoplasia*.

[58] Slezak-Prochazka I., Kluiver J., Jong D. D. (2016). Inhibition of the miR-155 target NIAM phenocopies the growth promoting effect of miR-155 in B-cell lymphoma. *Oncotarget*.

[59] D'Oro U., Munitic I., Chacko G., Karpova T., McNally J., Ashwell J. D. (2002). Regulation of constitutive TCR internalization by the *ζ*-chain. *Journal of Immunology*.

[60] Leidinger P., Backes C., Meder B., Meese E., Keller A. (2014). The human miRNA repertoire of different blood compounds. *BMC Genomics*.

[61] Alashti F. A., Minuchehr Z. (2013). MiRNAs which target CD3 subunits could be potential biomarkers for cancers. *PLOS ONE*.

[62] Sangokoya C., Telen M. J., Chi J.-T. (2010). microRNA miR-144 modulates oxidative stress tolerance and associates with anemia severity in sickle cell disease. *Blood*.

[63] Khan A. U., Rathore M. G., Allende-Vega N. (2016). Human leukemic cells performing oxidative phosphorylation (OXPHOS) generate an antioxidant response independently of reactive oxygen species (ROS) production. *EBioMedicine*.

